# Acetylcholine content and viability of cholinergic neurons are influenced by the activity of protein histidine phosphatase

**DOI:** 10.1186/1471-2202-13-31

**Published:** 2012-03-21

**Authors:** Anna Eißing, Daniel Fischer, Ilka Rauch, Anne Baumann, Nils-Helge Schebb, Uwe Karst, Karsten Rose, Susanne Klumpp, Josef Krieglstein

**Affiliations:** 1 Institut fuer Pharmazeutische und Medizinische Chemie, Westfaelische Wilhelms-Universitaet, Muenster, Germany; 2 Institut fuer Anorganische und Analytische Chemie, Westfaelische Wilhelms-Universitaet, Muenster, Germany

## Abstract

**Background:**

The first mammalian protein histidine phosphatase (PHP) was discovered in the late 90s of the last century. One of the known substrates of PHP is ATP-citrate lyase (ACL), which is responsible - amongst other functions - for providing acetyl-CoA for acetylcholine synthesis in neuronal tissues. It has been shown in previous studies that PHP downregulates the activity of ACL by dephosphorylation. According to this our present work focused on the influence of PHP activity on the acetylcholine level in cholinergic neurons.

**Results:**

The amount of PHP in SN56 cholinergic neuroblastoma cells was increased after overexpression of PHP by using pIRES2-AcGFP1-PHP as a vector. We demonstrated that PHP overexpression reduced the acetylcholine level and induced cell death. The acetylcholine content of SN56 cells was measured by fast liquid chromatography-tandem mass spectrometry method. Overexpression of the inactive H53A-PHP mutant also induced cell damage, but in a significantly reduced manner. However, this overexpression of the inactive PHP mutant did not change the acetylcholine content of SN56 cells significantly. In contrast, PHP downregulation, performed by RNAi-technique, did not induce cell death, but significantly increased the acetylcholine content in SN56 cells.

**Conclusions:**

We could show for the first time that PHP downregulation increased the acetylcholine level in SN56 cells. This might be a potential therapeutic strategy for diseases involving cholinergic deficits like Alzheimer's disease.

## Background

Reversible protein phosphorylation has become one of the most important mechanisms for regulating biological processes, maybe because of its simplicity and flexibility [1]. So far much is known about kinases and phosphatases that act on serine, threonine and tyrosine residues, but the knowledge about histidine phosphorylation is still fragmentary.

In the late 90s of the last century, the first mammalian histidine phosphatase was discovered by Hermesmeier and Klumpp [2] and described in detail by Klumpp et al. [3] and Ek et al. [4]. PHP is a small enzyme of 13.7 kDa that is ubiquitously expressed in mammalian tissues [3]. Structural investigations postulated histidine 53 to be essential for catalytic function of PHP [5,6].

Several proteins, as for instance P-selectin [7], histone H4 [8] and annexin I [9] are phosphorylated at a histidine residue and are essential for cellular signaling without knowing the kinase or phosphatase responsible for that. Meanwhile, three substrates have been identified for PHP i.e. ATP-citrate lyase (ACL) [10], the ß-subunit of the heterotrimeric G protein (Gß) [11] and the calcium-dependent potassium channel KCa3.1 [12].

Previous studies have shown that ACL regulates crucial parts of intermediate metabolism in neuronal cells and that PHP expression seems to impact ACL activity [13,14]. ACL is responsible for providing acetyl-coenzyme-A (acetyl-coA) for synthesis of fatty acids and of acetylcholine (ACh), i.e. the neurotransmitter which is used by cholinergic neurons and synthesized from choline and acetyl-coA. Therefore our present work focussed on the putative influence of PHP activity on ACh content of cholinergic neurons. It is already known that a decreased level of ACh is found in the brains of patients with Alzheimer's disease (AD) [15,16], but it is still unknown whether PHP plays a role in regulating the ACh level in brain tissue. In a previous study, we have developed a method for measuring the ACh content in cultured cholinergic neurons [17]. In the present work, we wanted to shed some light on the significance of PHP for the synthesis of ACh in cholinergic neurons. We investigated the viability of SN56 cells and measured the ACh content of these cholinergic neurons after up- and downregulation of PHP.

## Methods

### Materials

Dulbecco's modified eagle medium (DMEM), OptiMEM and gentamycine were obtained from Gibco (Eggenstein, Germany). Hoechst 33258, paraformaldehyde and α-tubulin antibody were from Sigma-Aldrich (Taufkirchen, Germany). Fetal bovine serum (FBS) and Trypsin-ethylenediaminetetraacetic acid (EDTA) for detaching SN56-cells were from PAA (Marburg, Germany). Horse radish peroxidase (HRP) conjugated antibodies were obtained from GE Healthcare (Freiburg, Germany).

### Monoclonal PHP antibody

Monoclonal antibody of PHP was used as previously generated and characterized [18].

### Cell culture and cell extracts

SN56.B5.G4 cells, a cholinergic murine neuroblastoma cell line, were provided by C. Culmsee (Marburg, Germany). The cells were cultured at 37°C in a humidified atmosphere containing 5% CO_2_. For cultivation of SN56 cells DMEM supplemented with 10% FBS and gentamycine (50 μg/ml) was used. Experimental procedures with SN56 cells were performed with passages 25 - 40. At confluency, cells were split 1:3-1:5 by incubation with trypsin/EDTA-solution. After washing cells were cultivated in 75 cm^2 ^flasks with 10^6 ^cells/flask.

Cell extracts for Western blot analysis were prepared by washing cells with ice-cold phosphate buffered saline (PBS; 13.7 mM NaCl, 2.7 mM KCl, 10 mM Na_2_HPO_4_, 1.8 mM KH_2_PO_4_) and were resuspended in homogenization buffer containing 50 mM Tris/HCl (pH 7.5), 1 mM EDTA, 0.1 mM phenylmethylsulfonyl fluoride (PMSF) and 1 mM benzamidine (BZ). For disruption cells were sonicated 3 times with 5 pulses (1/2 cycles and 60% amplitude; sonificator: Ultrason technology, Hielscher; Germany).

The total protein concentration was determined by using the Lowry assay [19] based on the protocol modified by Hartree [20].

### Immunoblotting

A total amount of 50 μg protein and 100 ng recombinant (rec.) PHP were applied on 15% SDS-PAGE gels. Proteins were transferred onto nitrocellulose membrane by semidry blotting for 1 h at 10 V and then the membrane was blocked with 5% non-fat milk powder in TBST. Monoclonal primary PHP antibody (1:200, 0.1% bovine serum albumin), α-tubulin (1:10,000, 5% non-fat milk powder) as a loading control and the appropriate secondary antibodies conjugated to HRP(1:2,500, Sigma) were used to accomplish immunodetection of PHP and α-tubulin. Membranes were incubated with the primary antibodies over night at 4°C and with the secondary antibodies for 1 h at room temperature (RT). Immunoreactive bands were visualized by using enhanced chemiluminescence (Thermo Sientific, IL, USA) and automatic film processor Cawomat 200 IR (AGFA, Germany).

### Transfection of SN56 cells

SN56 cells were washed with OptiMEM supplemented with 10% FBS and adjusted to 4,000,000 cells in 375 μl of the same medium. After adding 26 μg DNA or 25 μl of the siRNA solution (20 μM) in a 4 mm cuvette, electroporation was performed by using Genpulser Xcell (Bio-Rad; Munich, Germany). The cells from each transfection were seeded in a 6-well-plate for Western Blot analysis and ACh measurement. For Hoechst 33258 staining the transfected cells were seeded in 24-well-plates. Culture was achieved under standard conditions in DMEM containing 10% FBS, but no gentamycine.

### Overexpression of PHP

Mammalian PHP overexpression vector was constructed by inserting polymerase chain reaction (PCR) generated cDNA, using php-pET-16b [21] as a template, in pIRES2-AcGFP1 (Takara Bio Europe/Clontech; Saint-Germain-en-Laye, France). The primer with the sequence 5'-atc gga att cca tgg cgg tgg cgg a-3' was used as a forward primer and with the sequence 5'-cgg atc cgt cag tag ccg tcg tta gc-3' as a reverse primer for amplifying the PHP sequence with PCR. Both, PHP and green fluorescent protein (GFP) were expressed from a single bicistronic mRNA.

Site-directed mutagenesis of histidine 53 of the human PHP sequence cloned in pIRES2-AcGF1-PHP was inserted by PCR using oligonucleotides (H53Asense: 5'-gca gaa gtg ggc tga gta cgc cgc gga cat cta cg-3', H53Aanti: 5'-cgt aga tgt ccg cgg cgt act cag ccc act tct gc-3') of the mutated residue. The overexpression vectors were transferred into SN56 cells by electroporation as described under transfection of SN56 cells.

### Downregulation of PHP by siRNA

Transient downregulation of PHP was performed with chemically synthesized siRNA (Qiagen; Hilden, Germany). Several siRNA sequences were tested for their potency to downregulate PHP in SN56 cells. The siRNA corresponding to the nucleotides 5'-aac tga gaa gat caa agc caa-3' of the murine PHP sequence yielded best results. As negative control SN56 cells were transfected with scrambled siRNA. Transfection of SN56 cells with siRNA was performed as described in transfection of SN56 cells.

### Analysis of cell damage

For nuclear staining with Hoechst 33258 cells were seeded on 24-well-plates and cultured under appropriate conditions. The cells were washed with 37°C PBS, fixed for 30 min with 4% paraformaldehyd and then incubated with Hoechst 33258 (10 μg/ml) for 30 min at RT. Nuclear morphology was analyzed with the fluorescence microscope Axiovert 25 from Zeiss (Oberkochen, Germany).

### Acetylcholine measurement

Sample preparation for ACh measurement was performed as described previously [17]. In brief, SN56 cells were washed with PBS and were detached with trypsin-EDTA solution. Then trypsin was inhibited by 10% FBS in buffer wash solution (BWS). BWS contained 90 mM NaCl, 30 mM KCl, 20 mM 4-(2-hydroxyethyl)-1-piperazineethanesulfonic acid (HEPES), 0.02 mM EDTA, 32 mM sucrose, 0.015 mM neostigmine and 10% FBS, pH 7.4. For further preparation cells were washed twice with BWS (FBS free). Subsequently, cells for ACh measurement were resuspended in 80% acetonitrile containing 0.015 mM neostigmine and the cells for protein quantification taken from the same tube were resuspended in 10 mM HEPES (pH 7.4), 2 mM EDTA, 0.1 mM PMSF and 1 mM BZ. Acetylcholine content was measured by fast liquid chromatography-tandem mass spectrometry (LC-MS/MS) as described previously [17].

### Statistical analysis

All values are given as mean +/- standard deviation. Significant differences between the means were calculated by analysis of variance (ANOVA) followed by Scheffé-test.

## Results

### Overexpression of PHP and cell damage

**Figure 1:**
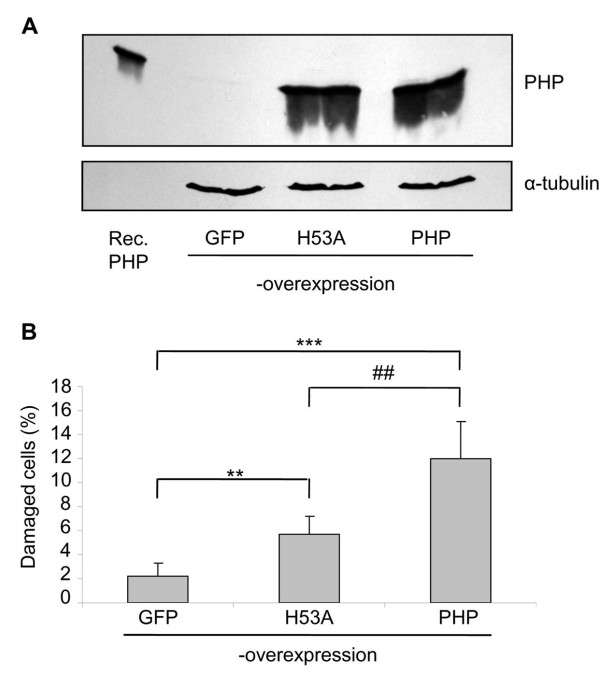
**Overexpression of PHP in cholinergic neurons causes cell damage**. (A) Western blot analysis shows overexpression of PHP and of its inactive mutant in SN56 cells after 48 h. An amount of 100 ng recombinant PHP and 50 μg total cell lysate were applied for SDS-PAGE. PHP was detected by the monoclonal PHP antibody. As a loading control α-tubulin was used and detected by the monoclonal α-tubulin antibody. (B) Percentage of damaged SN56 cells was determined by Hoechst 33258 staining and was increased after PHP overexpression. However, cell damage was less pronounced after overexpression of the H53A-PHP mutant. Values are given as means ± standard deviationof 6 experiments. ***p < 0.001 and **p < 0.01 compared to GFP-overexpression; ^##^*p *< 0.01 compared to H53A-overexpression (ANOVA followed by Scheffé-test)

Overexpression of PHP in SN56 cells was performed by using pIRES2-AcGFP1-PHP as a vector. A time-dependent analysis of PHP overexpression showed that best results were achieved 48 h after transfection with the PHP-overexpression vector (data not shown). As shown in Figure 1A PHP is overexpressed in SN56 cells by using pIRES2-AcGFP1-PHP vector. In contrast, when using a GFP-overexpression vector PHP was indeed not overexpressed. To exclude unspecific effects of protein overexpression, the inactive histidine 53 mutant of PHP was overexpressed, too.

The influence of overexpression of PHP on cell viability was analysed by Hoechst 33258 staining. PHP overexpression for 48 h significantly caused damage of SN56 cells of about 12% in comparison to the negative control of GFP overexpression (Figure 1B). Inactive H53A-PHP overexpression did also cause cell damage of about 5%, but in a significantly reduced manner compared to wt-PHP overexpression.

### PHP overexpression and acetylcholine level

**Figure 2:**
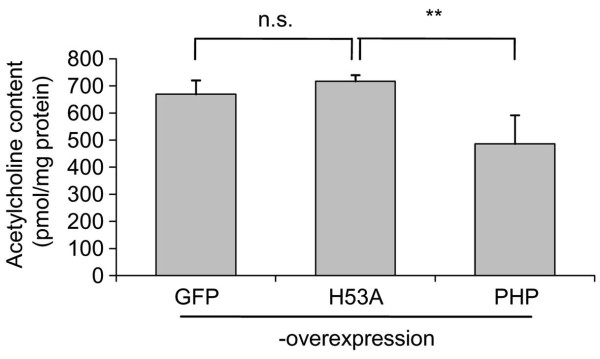
**Acetylcholine content after PHP overexpression**. To measure the ACh content of SN56 cells, the cells were harvested 48 h after transfection with the overexpression vectors and ACh was measured by LC-MS/MS. Values are given as means ± standard deviation of 4 experiments. **p < 0.01 compared to H53A-overexpression; n.s. (not significant) compared to GFP-overexpression (ANOVA followed by Scheffé-test). Additionally, *p *< 0.01 between GFP- and PHP-overexpression was determined

Previous studies showed that PHP dephosphorylates ACL and therefore diminishes ACL activity [13,14]. ACL is essential for providing acetyl-CoA for ACh synthesis [16]. We measured the ACh content of SN56 cells 48 h after transfection with the PHP overexpression vector. It is shown in Figure 2 that PHP overexpression decreases significantly the ACh content of SN56 cells from about 700 to about 500 pmol/mg protein. To exclude negative effects of raised PHP protein content itself, the inactive H53A-PHP mutant was overexpressed, too. But the overexpression of the inactive PHP mutant did not cause a decrease of ACh content of SN56 cells compared to control (GFP-overexpression).

### PHP downregulation and cell viability

Our data suggest that PHP overexpression induces cell damage. As shown before the decreased cell viability correlates with a decreased phosphorylation state of ACL [14]. Furthermore, the ACh content of SN56 cells was significantly reduced after PHP overexpression (Figure 2).

**Figure 3:**
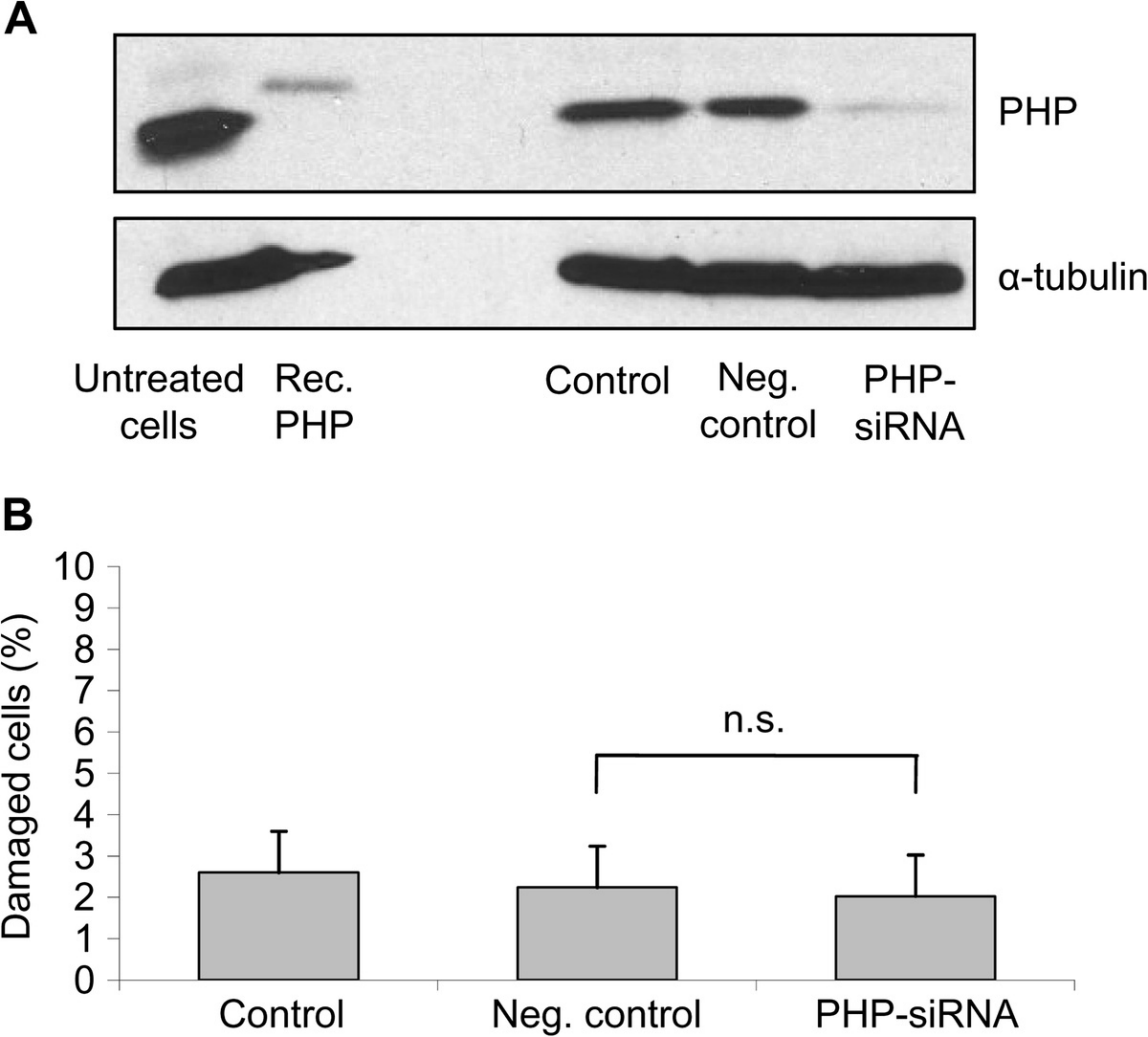
**Downregulation of PHP in cholinergic neurons and its impact on cell damage**. *Control*: SN56 cells transfected without siRNA. *Negative (Neg.) control*: SN56 cells transfected with scrambled siRNA. *PHP-siRNA*: transfected with siRNA against PHP. (A) PHP downregulation could be detected by Western blot analysis 48 h after transfection. An amount of 100 ng rec. PHP and 50 μg total cell lysate were applied for SDS-PAGE followed by Western blot analysis. PHP was detected by the monoclonal PHP antibody. As a loading control α-tubulin was used and detected by the monoclonal α-tubulin antibody. (B) Hoechst 33258 staining shows that PHP downregulation did not significantly induce cell damage after 48 h. Values are given as means ± standard deviation of 8 experiments. n.s. compared to neg. control (ANOVA followed by Scheffé-test)

To test the causal role of PHP in these processes, we downregulated PHP with RNAi-technique and analysed the influence of PHP downregulation on the viability and the ACh content of SN56 cells. Previous experiments showed that PHP is almost completely downregulated after 48 h of treatment with siRNA against PHP. Western Blot analysis showed that PHP is significantly downregulated 48 h after treatment with siRNA against PHP in comparison to the negative control (Figure 3A). Viability of SN56 cells was tested by staining with Hoechst 33258 at 48 h after transfection with siRNA against PHP. In line with PHP downregulation in other neuronal cells [13] cell viability was not affected after PHP downregulation (Figure 3B). In contrast to PHP overexpression which damaged SN56 cells, PHP downregulation did not affect cell viability at all.

### Acetylcholine level after PHP downregulation

**Figure 4:**
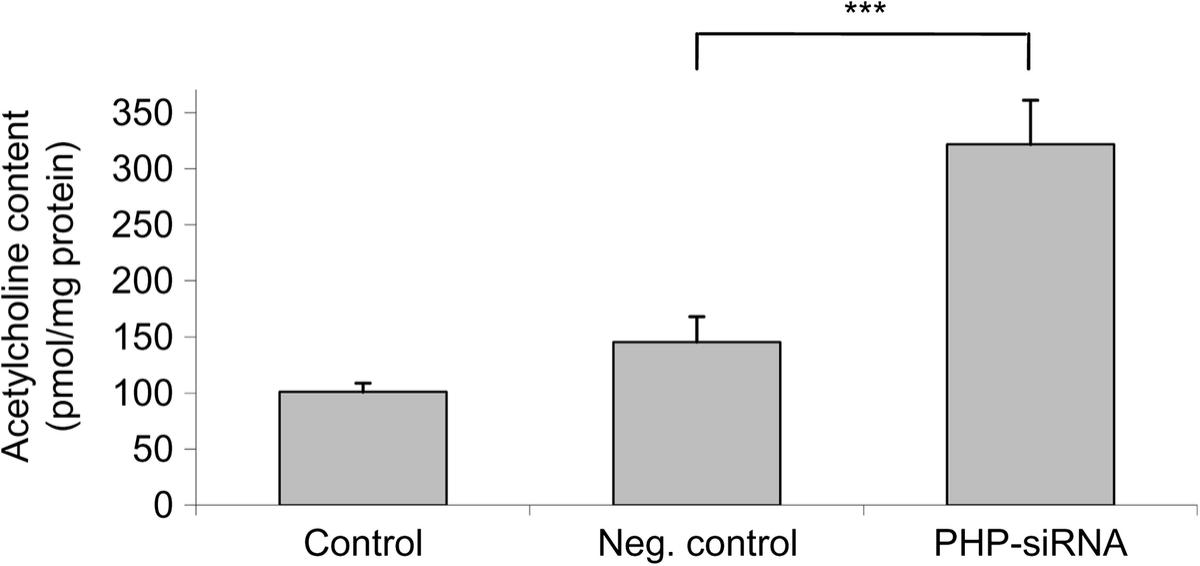
**Acetylcholine content and PHP downregulation**. SN56 cells were transfected with siRNA. After 48 h of siRNA treatment cells were harvested for ACh measurement. Values are given as means ± standard deviation of 4 experiments. ***p < 0.001 compared to neg. control (ANOVA followed by Scheffé-test)

In this work we wanted to find out whether PHP downregulation could modify the ACh content of SN56 cells. Therefore, we downregulated PHP for 48 h with the RNAi-technique and measured the ACh content of these SN56 cells by LC-MS/MS according to [17]. A significantly increased ACh content of SN56 cells was detected after PHP downregulation in contrast to the negative control where scrambled siRNA was used and PHP was not downregulated (Figure 4). The ACh content of SN56 cells increased from about 150 to 350 pmol ACh/mg protein after PHP downregulation. Obviously, there is a correlation between PHP downregulation and an increased ACh level in SN56 cells.

## Discussion

One of the most important substrates of PHP known so far is ACL [10]. PHP regulates the phosphorylation state and thereby the activity of ACL. ACL activity is decreased by PHP-catalyzed ACL dephosphorylation and increased by ACL phosphorylation. Downregulation of PHP expression causes less dephosphorylation of ACL and probably maintains a higher activity of this enzyme.

ACL is a cytosolic enzyme which cleaves citrate transported out from the mitochondria into acetyl-CoA and oxaloacetate [22]. Acetyl-CoA is used in cytosol for fatty acid and ACh synthesis whereas oxaloacetate is taken up by the mitochondria again and may stimulate energy metabolism. Beside the acetylcholine precursor synthesis via ACL, biosynthesis of the neurotransmitter ACh is dependent on both the regulated, rate-limiting choline uptake step and choline acetyltransferase-catalyzed linking of choline and acetyl-coA [22]

In previous experiments we could show that an increased expression of PHP leads to damage of neurons and endothelial cells [13,14,18]. Our hypothesis was that higher content of PHP in the cells means a higher dephosphorylation rate of PHP substrates like ACL. On the other hand, a lowered ACL activity leads to a reduced acetyl-CoA production and, consequently, to a reduced ACh content as demonstrated in the present paper. In addition, the reduced fatty acid and energy metabolism after PHP overexpression [13] could essentially contribute to the decreased viability of SN56 cells found in our study.

Taken together, overexpression of PHP is disadvantageous for neurons whereas downregulation of PHP synthesis increases the ACh content of cholinergic cells and, at least, does not negatively influence cell viability. PHP-H53A was overexpressed in SN56 cells and the increase of this inactive protein does influence the viability of SN56 cells slightly, but in a significantly reduced manner compared to wt-PHP overexpression. The ACh content of the cholinergic cells was not affected by this inactive PHP mutant.

Inhibition or downregulation of PHP seems to be a highly interesting strategy for treatment of AD. The brains of AD patients show indeed decreased ACh [23] and glucose contents [24]. To increase the ACh amount of AD brains by ACh esterase inhibitors is the most effective therapy of AD at the moment [25]. Decrease of PHP activity could cause not only an increase of ACL activity and consequently ACh content but also an increase of energy metabolism in neuronal cells. In other words, PHP inhibition might improve the damaged transmission of cholinergic neurons and, additionally, support their viability.

In conclusion, PHP could be an appropriate target for AD therapy because downregulation of PHP activity leads to an increase of ACL content as well as fatty acids and energy metabolism. Thus, functional activity and viability of cholinergic neurons might be improved by targeting PHP.

## Abbreviations

PHP: Protein histidine phosphatase; ACL: ATP-citrate lyase; siRNA: Small interfering RNA; ACh: Acetylcholine.

## Authors' contributions

AE carried out the experiments with neuronal cells and helped draft the manuscript; DF and IR carried out experiments with neuronal cells; AB and NHS carried out experiments to analyse acetylcholine content; UK participated in method development of acetyl choline analysis; KR participated in experiments with neuronal cells and helped draft the manuscript; SK and JK designed the study and wrote the manuscript. All authors read and approved the final manuscript.
